# Poverty and Quality of Life Dimensions: A Cross-Sectional Study of Diabetic Patients in Morocco

**DOI:** 10.3390/healthcare13070725

**Published:** 2025-03-25

**Authors:** Aicha El Hanafi, Safiya Mahlaq, Mohamed Lmejjati

**Affiliations:** 1 REGNE Research Laboratory, Faculty of Medicine and Pharmacy of Agadir, Ibn Zohr University, Agadir P.O. Box 8106, Morocco; 2 Laboratory of Biostatistics, Clinical Research and Epidemiology (LBRCE), Faculty of Medicine and Pharmacy of Rabat, Mohammed V University, Rabat 10000, Morocco

**Keywords:** quality of life, poverty status, socioeconomic level, diabetes, Morocco

## Abstract

**Background:** The assessment of quality of life highlights the effects of diabetes on patients. While the disease’s impact is often similar, socioeconomic conditions lead to notable differences. The objective of this study is to determine the impact of poverty status on the dimensions of quality of life of patients with diabetes. **Materials and Methods:** We conducted a cross-sectional analytical study among diabetic patients in the province of Agadir Ida Outanane, Morocco. We measured quality of life using the Moroccan version of the D 39 diabetes-specific quality of life questionnaire. Multiple linear regression was applied to predict the relationship between poverty status and quality of life dimensions, with adjustments for other covariates (sociodemographic, clinical, and therapeutic); univariate analyses were significant with a *p* < 0.25 value and multiple linear regression at *p* < 0.05. **Results:** There were 338 confirmed diabetic patients undergoing treatment included in the study. The poverty rate among this diabetic population was 37.3%. The study revealed median scores for impaired quality of life in diabetes control 62.5 [50.5–75], anxiety and worry 81.3 [56.3–93.8], overall perceived quality of life 50 [25–50], and diabetes severity 75 [50–100]. The results of multiple linear regression demonstrated that poverty status was associated with both dimensions anxiety and worry (β = 13.95, IC 95%: 8.12, 19.78, *p* < 0.001) and diabetes control (β = 8.90, IC 95%: 4.82, 12.97, *p* < 0.001). **Conclusions**: The management and psychological impact of diabetes are influenced by poverty status. It is crucial to prioritize this vulnerable population to enhance the effectiveness of interventions for comprehensive disease management.

## 1. Introduction

Global leaders have prioritized diabetes as one of the four most important non-communicable diseases, making it a significant public health problem. In recent decades, there has been a consistent rise in the prevalence of diabetes and the number of cases of the disease [1]. According to the World Health Organization estimates (2021), the situation in Morocco is similar, with a prevalence rate of 12.4% among adults. Annually, the disease is responsible for more than 12,000 deaths, with a further 32,000 deaths occurring due to complications [2].

This chronic metabolic disease significantly affects an individual’s overall well-being and social interactions [3], manifesting in a wide range of effects on the physical, social, and psychological aspects of health [4]. Both type 1, as well as type 2, diabetes are determinants of health outcomes, including health-related quality of life (HRQoL) [5].

Diabetes requires a series of lifestyle modifications, frequent monitoring, and adherence to a strict treatment regimen. These conditions, along with the potential acute and chronic complications of diabetes, can impact a patient’s physical and mental well-being, thereby affecting their quality of life (QoL) [6].

For chronic diseases such as diabetes, HRQoL is a key outcome due to the lifelong nature of the disease and the need for daily self-management [7]. Quality of life is an essential health outcome in its own right, representing the ultimate goal of all healthcare interventions. Therefore, its measurement should be systematically integrated into both clinical practice and research in the field of diabetes treatment [8].

Certainly, the mere presence of diabetes deteriorates a person’s health, and when diabetes coexists with other conditions, the effect is even more detrimental. Although the disease affects the main components of quality of life, differences in socioeconomic status and lifestyle habits exist [9]. Social classes are associated with varying health and disease outcomes [10]. Several publications and studies have linked poor socioeconomic status to health and disease. Recent studies have shown that poverty is the most important factor influencing diabetes [11]. Additionally, poor economic status is significantly associated with poor quality of life [12]; it is a significant determinant of poor QoL [13,14].

The extant literature has identified studies that demonstrate the relationship between poor socioeconomic status and QoL. Nevertheless, the majority of these studies have compared it in relation to overall quality of life and have examined it with generally associated factors. The aim of this study is, first, to measure specific aspects of quality of life in people with diabetes in order to highlight the dimensions that are impaired and, second, to explore and detail the relationship between living in poverty and the different dimensions of QoL in people with diabetes. The study points to the need to consider socioeconomic conditions in order to better manage diabetes and improve quality of life in these different dimensions.

## 2. Materials and Methods

### 2.1. Study Design

This study is an analytical, cross-sectional, and observational research of diabetic patients receiving care at both urban and rural primary healthcare centers in the province of Agadir Ida Outanane, located in the Souss Massa region of Southern Morocco. The study was conducted in the year 2023.

The inclusion criteria were as follows: diabetic patients diagnosed and treated for type 1 or type 2 diabetes for at least 6 months and diabetic patients aged 18 years and older. The exclusion criteria were as follows: women with gestational diabetes, diabetic patients not attending primary healthcare centers, diabetic patients with a mental illness that limits their intellectual capacity, and diabetic patients of foreign nationality.

The total sample size was 338 patients. The website OpenEpi [15], which calculates sample sizes, served as the basis, for a total diabetic population of 18,149 individuals followed up at primary healthcare centers. This calculation was based on a margin of error of 5%, a confidence level of 95%, and a national relative poverty rate of 12.7%.

Referring to the statistical data concerning diabetic patients in the province, the patients were recruited according to the quota sampling modality based on urban (74%) and rural (26%) areas. These patients were selected for participation during their follow-up visits or while receiving treatment at the chronic disease units. The study involved six centers that have the largest populations of diabetic patients in the province. All eligible diabetic patients who attended these centers during the study period were consecutively enrolled until the minimum required sample size was achieved.

### 2.2. Data Collection and Measurement Instruments

The collected information covered sociodemographic, clinical, therapeutic, and health-related quality of life (HRQoL) characteristics of diabetic participants. A questionnaire was crafted to compile the key variables reported in the literature on QoL studies involving diabetic patients [16,17,18].

The sociodemographic characteristics were as follows: age, sex, residence area, marital status, occupational status, educational level, poverty status, and health insurance coverage. The clinical data collected included duration of diabetes in years; most recent glycated hemoglobin level (HbA1c) (<7% ‘optimal’, between 7% and 9% ‘suboptimal’, and >9% ‘high risk’); comorbidity; hypertension comorbidity; chronic complications; obesity; and smoking status. Therapeutic information included the following: adherence to treatment; type of treatment used; frequency of medical visits per year (<4, 4 “recommended”, or >4); specialty of treating physician; and use of traditional treatment.

The measurement of poverty situation, which is the basic independent variable for this study, is based on the criterion of household income. This criterion has been conceptualized as the fact of living in poverty, as set by the Moroccan High Planning Commission at 50% of the median household income [19]. This method is the most appropriate for measuring this multidimensional variable, and it is the method employed by the majority of Moroccan population surveys.

Health insurance coverage: In Morocco, Law 65-00 on the Basic Medical Coverage Code established two coverage plans: an obligatory health insurance (AMO) plan for employees and retirees in the public and private sectors, as well as their dependents, and a medical assistance plan (RAMED) for economically disadvantaged individuals. RAMED is based on the principles of social assistance and national solidarity and is exclusively applicable for patient care in public health facilities [20]. At present, following the 2022 reform as part of the social protection project, a new obligatory health insurance scheme (AMO Solidarity) was created. This scheme replaces the ‘RAMED’ system and provides health insurance to households that cannot afford the contributions, especially those previously under RAMED [21]. Since this study was conducted in 2023, a transition year, some patients continued to benefit from their ‘RAMED’ insurance while awaiting the full implementation of AMO Solidarity. Therefore, the categorization used for presenting this aspect of medical coverage was None, ‘RAMED’/AMO ‘Solidarity’, and AMO.

Treatment adherence was measured using the Arabic version of the Morisky, Green, and Levine (MGL) adherence scale, which was adapted and validated by Awwad et al. in 2022 [22]. This instrument consists of four questions aimed at identifying instances of medication non-adherence among patients with chronic diseases, such as diabetes. This Arabic version of the Morisky, Green, and Levine scale has demonstrated its reliability and validity, exhibiting psychometric properties similar to those of the original English version [22]. The scale uses a simple scoring system, with ‘Yes’ = 1 and ‘No’ = 0 for each item. Lower scores indicate higher levels of adherence. Patients’ total scores are then classified into three categories: high adherence (answering ‘yes’ to 0 items), moderate adherence (answering ‘yes’ to 1 or 2 items), and low adherence (answering ‘yes’ to 3 or 4 items) [23].

Clinical characteristics regarding the treatment used and the most recent HbA1c level were reported from patients’ follow-up records. Obesity status was calculated according to the body mass index (BMI) using the Quetelet formula (weight (kg)/height^2^) and defined according to the World Health Organization (WHO) classification: obesity ≥ 30 kg/m^2^ [24].

Chronic complications included eye disease, heart disease, kidney disease, foot disease (neuropathy and diabetic foot), and comorbidity (other associated chronic diseases). The information was either self-reported by the patient or obtained from the follow-up record, if available.

With regards to health-related quality of life (HRQoL) in diabetic patients, the specific scale on quality of life in diabetic patients, D 39 Moroccan Arabic version, adapted and validated by Adarmouch et al. in 2020, was used to collect data on quality of life [25]. A specific scale was chosen for this study, because it allows for a better identification of the specific problems associated with the disease [26].

The D 39 is a diabetes-specific self-report questionnaire developed in the USA in 1997 by Boyer and Erap. It is a quality of life scale designed for patients with type 1 and type 2 diabetes. The questionnaire consists of 39 items divided into five dimensions: energy and mobility (15 items: 3, 7, 9, 10, 11, 12, 13, 16, 25, 29, 32, 33, 34, 35, and 36); diabetes control (12 items: 1, 4, 5, 14, 15, 17, 18, 24, 27, 28, 31, and 39); social limitations (5 items: 19, 20, 26, 37, and 38); anxiety and worry (4 items: 2, 6, 8, and 22); and sexual functioning (3 items: 21, 23, and 30). The D 39 questionnaire also included two individual items: an overall self-assessment of QoL and a self-assessment of diabetes severity [27,28,29].

A request and agreement for the use of the Moroccan dialect version of the D 39 were obtained from the developers of the instrument. The patients were then asked to evaluate the quality of life experienced over the past month for each item on a scale of 1 to 5 [25]. The study used a generic formula to transform the scores for each dimension, perceived QoL, and diabetes severity on a scale from 0 to 100: (gross classification − minimum value)/(maximum value − minimum value) × 100. This formula normalizes any values between upper and lower limits, ensuring consistent linear transformation across various dimensions [28].

The patients’ assessment yields scores ranged from 0 to 100, providing a quantitative measure of their QoL dimensions. Scores tending towards 100 indicated an altered QoL. On the other hand, a score tending towards 0 indicated a better QoL, except for the scores for overall perceived quality of life, which were reversed. A study of the psychomotor characteristics of the Moroccan version of the D 39 showed good validity and reliability of this adapted questionnaire [25].

Questionnaire data collection was conducted through face-to-face interviews in a neutral and non-suggestive manner with people with diabetes due to high rates of limited education. The average time required to complete the two questionnaires was approximately 15 to 20 min.

### 2.3. Statistical Analysis

A descriptive analysis was conducted to present sociodemographic, clinical, therapeutic, and QoL data. Categorical variables were presented as numbers and percentages, while continuous variables were presented as the mean ± standard deviation for normally distributed variables and as the median and interquartile range for asymmetrically distributed variables. The normality of the variables was assessed through the implementation of the Shapiro–Wilk test. Depending on the utilization conditions and the characteristics of the data, either parametric or non-parametric tests were employed for continuous variables.

For each dimension that demonstrated a significant association with poverty, a univariate linear analysis was conducted with the other sociodemographic, clinical, and therapeutic variables present in the study. Subsequently, only those variables found to be significant in the univariate analysis were incorporated into a standard multiple linear regression analysis. The objective was to identify the predictors of QoL for the specified dimensions and to verify the adjusted associations between poor socioeconomic level and QoL after controlling for other variables.

The standard significance level was set to 0.05. The significance level for variables entered into the multiple regression model was set at 0.25. All statistical procedures were performed with jamovi (version 2.3.28).

### 2.4. Ethical Considerations

All participants were informed of the objectives of the study. Informed consent was obtained from all participants. They were assured that their personal information would remain confidential and anonymous. The study was approved by the Ethics Committee for Biomedical Research of the Faculty of Medicine and Pharmacy of Rabat Mohammed V University (File number 16/23).

## 3. Results

### 3.1. Sociodemographic, Clinical, and Therapeutic Characteristics of the Patients

The study population comprised 338 participants, of whom 62.1% were female. The median age of the participants was 55 [45–64], with extremes of 20 and 83 years. The majority (68.3%) were married, and nearly half (48.5%) had no formal education. Most participants (68%) were not employed, and 74% resided in urban areas. The majority had health insurance coverage, which was distributed as follows: 41.8% the obligatory health insurance (AMO) and 38.6% ‘RAMED’ or the obligatory health insurance ‘Solidarity’ for the economically disadvantaged population (Table 1).

The poverty rate among the diabetic population under study was 37.3% (Table 1).

Regarding of the clinical characteristics, 40.5% of patients had a comorbidity, and 32% had a specific hypertension comorbidity. Half (51.2%) had chronic diabetic complications, 6.5% were obese, and 7.7% were smokers. In terms of glycated hemoglobin HbA1c control, the majority (70.4%) had suboptimal control levels ranging from 7% to 9%. Furthermore, the data indicate that 51.8% of the patients had been diagnosed with diabetes for a period of 10 years or less (Table 2).

The therapeutic data are as follows: A significant proportion of the diabetic participants (66.6%) had better adherence to treatment, and nearly half (49.4%) had medical follow-up of less than the recommended four visits per year. Almost half of the patients (45.9 were treated solely with insulin. Half (50.3%) were treated by a general practitioner, and 17.5% used a traditional treatment in addition to medical treatment (Table 2).

### 3.2. Health-Related Quality of Life for Patients Living in Poverty

The mean scores for each dimension were as follows: energy and mobility 48.3 [33.3–70], diabetes control 62.5 [50.5–75], anxiety and worry 81.3 [56.3–93.8], social limitations 25 [10–35], and sexual functioning 25 [0–64]. The mean score for the item used to summarize participants’ overall QoL was 50 [25–50], and the mean score for the item used to summarize diabetes severity was 75 [50–100]. The analysis of the median scores impaired four dimensions: diabetes control, anxiety and worry, overall perceived QoL, and the severity of diabetes (Table 3).

Cronbach’s alpha for the scores of energy and mobility, diabetes control, anxiety and worry, social limits, and sexual functioning was 0.92, 0.86, 0.83, 0.70, and 0.94, respectively, demonstrating the good reliability of D 39 used in our study (Table 3).

### 3.3. The Association Between Poverty Status and Health-Related Quality of Life Dimensions

A statistical analysis was conducted to predict the association between living in poverty and the QoL of diabetic patients. The analysis was performed for each dimension of D 39 and poverty status (see Table 3).

Living in poverty has an impact on the HRQoL of diabetic patients. Indeed, the significant dimensions of this analysis are diabetes control (*p* ≤ 0.001), anxiety and worry (*p* ≤ 0.001), sexual functioning (*p* =< 0.001), and perceived global HRQoL (*p* = 0.011).

Sexual functioning showed a significant association in the univariate analysis but was excluded from the multiple linear regression model because of missing data in this dimension. Missing data can reduce the statistical power of a study and produce biased estimates, leading to invalid conclusions [30]. The presence of missing data for this dimension of sexual functioning is linked to the non-response of certain participants due to their sexual inactivity or their refusal to respond. As other authors have noted, this reluctance to respond may be attributable to the fact that sexuality is a taboo subject in the Arab context. Questions pertaining to this subject are often perceived as sensitive, delicate, and personal [25,31].

Initially, univariate analysis was employed to identify predictors of QoL for the dimensions that showed significance with poverty status (Table 4). Multiple linear regression analyses were then used to verify the adjusted associations between poverty status and these dimensions after adjusting for other confounding variables that revealed a significance, as shown in Table 4.

Multivariate analysis revealed that a poverty status is associated with both dimensions anxiety and worry and diabetes control (β = 13.95, IC 95%: 8.12, 19.78, *p* < 0.001 and β = 8.90, IC 95%: 4.82, 12.97, *p* < 0.001), respectively (Table 5).

## 4. Discussion

The current global recommendation is to adapt diabetes management to risk and QoL, which is a critical element in determining the success or failure of diabetes management [32]. Moreover, diabetes is a condition where active patient involvement in disease management is crucial. Therefore, focusing on the psychosocial aspects and experiences of people with diabetes was the focal point of the rationale behind the cross-national Diabetes Attitudes, Wishes and Needs study (DAWN) [33]. This study aims to align with both recommendations and, as such, has made it possible to report new information on the QoL of people with diabetes, particularly those living in poverty. To our knowledge, no study has assessed the relationship between poor socioeconomic status and dimensions of QoL in diabetic patients in Morocco. The results of previous studies indicate that efforts to improve the QoL of people with chronic diseases cannot succeed without addressing the social determinants of health [34].

Our results showed a poverty rate of 37.3% among this diabetic population, which is higher than the national relative poverty rate for the Moroccan population, which is 12.7%. This could be explained by the increased prevalence of diabetes among socioeconomically disadvantaged individuals—that is, people with lower income levels [35]. Additionally, the study was conducted in public health centers, which justifies the proportion of the poor population using these services to access care.

According to the results of the QoL of patients with diabetes, median scores representing impaired QoL were presented for the following dimensions: diabetes control 62.5 [50.5–75], anxiety and worry 81.3 [56.3–93.8], overall perceived QoL 50 [25–50], and diabetes severity 75 [50–100].

These results can be compared with certain limited studies of QoL in diabetic patients using the same scale, the D 39. In fact, the results are similar to a study of young Jamaican diabetics, which also used the D 39 questionnaire. That study also scored highest on the diabetes control and anxiety and worry subscales and lowest on the social burden and sexual functioning scales [36]. A study conducted in Morocco also found that the anxiety and worry dimension had the highest median score (60.4) compared to the other QoL dimensions [25]. Similarly, two other studies, one conducted in Nepal and a second in Peru, showed the same results for the dimension of anxiety and worry, with median scores of 54.2 and 66.7, respectively [37,38]. The current findings suggest that diabetes affects the psychological aspect of QoL. Addressing this dimension is crucial to preventing mental health issues, such as depression related to diabetes [39].

In our study, the diabetes control dimension was also highly significant (median score of 62.5). This is in contrast to the findings of a study conducted in Morocco using the D-39 scale, which had a median score 44.5 [25]. A comparable finding was reported in another study, which indicated that the diabetes control dimension, with a median score of 46.6, achieved the highest median score among all the QoL dimensions [40]. The same study found a median diabetes severity score of 66, which is comparable to the results of our study, with a median score of 75. This result is further supported by a study conducted in 2021, in which the median score was 50 [37]. The overall perceived quality of life in the present study was improved compared to the other studies (median score of 50) [40,41].

The average scores for each dimension in our study were slightly higher compared to other studies. This difference in median scores across various dimensions can be attributed to differences in living conditions, cultural orientation, and healthcare systems between countries and regions. As noted by other researchers, specific factors such as socioeconomic status, clinical conditions, and paraclinical elements likely influenced these results. Therefore, it is essential to consider these indicators when evaluating study results [42].

Regarding the association between poor socioeconomic status and quality of life in diabetic patients, the extant literature generally indicates that income and social status are significant predictors of QoL in diabetic patients [43].

The results of multiple linear regression showed that being from a poor household affected QoL for two dimensions: anxiety and worry (β = 13.95, IC 95%: 8.12, 19.78, *p* < 0.001) and diabetes control (β = 8.90, IC 95%: 4.82, 12.97, *p* < 0.001). Consistent with these findings, one study revealed higher average scores, indicating a greater impact on both domains for participants with lower incomes [26].

About anxiety and worry, a 2024 global survey by the International Diabetes Federation (IDF) revealed that 77% of people with diabetes have experienced anxiety, depression, or another mental health disorder due to their condition [44]. Researchers have endeavored to identify and explain the sources of psychological distress, linking it to the ongoing burden and constant demands of managing the disease, as well as the fear of complications. Diabetes-related distress includes emotions such as guilt, anxiety, and concerns about self-management of the condition [45,46].

As indicated by the findings in the present study, the mental dimension score for anxiety and worry increases in individuals living in poor socioeconomic conditions (β = 13.95, IC 95%: 8.12, 19.78, *p* < 0.001), indicating an alteration in this component, in accordance with other studies on both type 1 and type 2 diabetes, which indicate that a higher income is a significant predictor of better QoL in terms of anxiety and worry [47]. A higher social status was also significantly associated with more favorable scores in various domains of QoL, including worry about the future [48]. Moriris and Chasens asserted that participants experiencing moderate to extreme financial difficulties reported poorer mental quality of life and more diabetes-related psychological distress (all *p*-values < 0.05) compared to those without financial difficulties, thereby exacerbating barriers to self-care [49]. In general, a poor level of economic status is one of the factors that predicts a lower quality of mental life [50].

Referring to the results of this investigation, poverty has emerged as a significant factor contributing to the deterioration of the dimension related to diabetes control in QoL. There was a significant positive association between poverty and diabetes control (β = 8.90, IC 95%: 4.82, 12.97, *p* < 0.001). Studies have reported a higher risk of suboptimal control (OR 2.27, *p* < 0.001) in the lowest quartile of socioeconomic status [51]. Similarly, another study concluded that socioeconomic status significantly influenced glycemic control (*p* = 0.005), with 75% of patients from lower socioeconomic classes having uncontrolled HbA1C levels [52]. Another investigation found contributing factors that often coexist with poverty, such as food insecurity, disparities in access to healthcare, and related mental health problems. These factors hinder the adoption of lifestyle changes that are crucial for managing diabetes, particularly type 2 [53]. The impact of the socioeconomic situation of poverty on diabetes control in a developing country could result from the total financial cost of the disease, with expenses ranging from medication to blood glucose monitoring and frequent laboratory testing, as diabetes care is not completely free in these countries [52]. This is equally applicable to our context in Morocco.

It should be noted that, despite the significant proportion of patients in this study having medical coverage. The program ‘RAMED’, previously introduced for the poor population, had significant flaws and failed to produce the desired results [54], especially when households continued to pay for health services. [55]. Also, even when benefiting from AMO or AMO ’Solidarity’ health coverage (social protection reform to replace RAMED), poor patients report challenges in accessing private healthcare services that require upfront payments or copayments. These copayments are higher in the private sector compared to the public sector [56]. These factors affect access to healthcare and, consequently, the control of diabetes. In addition, other authors have confirmed that, for the non-poor population, a higher socioeconomic status can reduce the financial burden of diabetes and facilitate access to better quality medical care. A higher socioeconomic status also fosters better nutrition, as well as access to specialized healthcare and counseling, which can have a significant impact on disease control [26,57,58].

## 5. Strengths and Limitations of the Study

The study’s primary strength is its focus on specific dimensions of HRQoL influenced by poor socioeconomic status. This focus will assist in elucidating the aspects necessitating attention in the management of diabetes among precarious populations, particularly Moroccans. Another strength is the use of the Moroccan version of the D 39, a specific, valid, and reliable instrument for measuring the QoL of diabetics, as opposed to other studies that have used generic questionnaires. Thirdly, stratification by urban and rural residences enabled the sample size to be representative of populations from both areas.

The limitations of this study are as follows: (a) the measurement of the ‘poverty’ variable only by the relative poverty index (in relation to household income), (b) the low prevalence of the independent variable ’poverty’ in the studied population may restrict the generalizability of the results, and (c) other variables (related to patients’ knowledge and behaviors regarding their illness) that could be classified as confounding variables were not considered in this study.

## 6. Conclusions

In Morocco, diabetes is increasingly becoming a significant clinical burden. The majority of patients do not achieve the recommended objectives. Based on this research, the dimensions of anxiety and worry, as well as diabetes control, were found to be the only ones associated with poverty status within the context of QoL. It is recommended that the following measures be taken to remedy the situation. Although Morocco provides quality care in terms of treatment supply for the vulnerable diabetic population, this remains insufficient. This is due to the emergence of other care needs that affect the overall care process. Reducing disparities that hinder equitable access to specialized healthcare remains an objective to be attained.

In addition, patient involvement in the management of their disease should be reinforced through therapeutic education that considers the patient’s living environment (resources, eating habits, and prioritization). This education is essential to facilitate and promote healthy behaviors. Furthermore, the improvement and restructuring of medical insurance coverage will help reduce the financial burden of diabetes care and its complications for populations living in poverty.

The emotional and psychological needs of diabetic patients living in poverty are emphasized. This underscores the importance of reinforcing the essential roles of general practitioners, diabetologists, and healthcare professionals, and particularly mental health professionals, in mitigating and managing the psychological difficulties faced by these patients, which are exacerbated by their socioeconomic conditions. In conclusion, the intervention approach is multidisciplinary, requiring the mobilization of the efforts of all those involved, including the patients themselves.

## Figures and Tables

**Table 1:** Sociodemographic characteristics.

Variables	Participants n = 338 (N%)
Gender:	
Male	128 (37.9)
Female	210 (62.1)
Age (years) (median and IQR)	55 [45–64]
<60 ans	192 (56.8)
>=60 ans	146 (43.2)
Living area:	
Rural	88 (26)
Urban	250 (74)
Marital status:	
Single	36 (10.7)
Married	231 (68.3)
Divorced	22 (6.5)
Widowed	49 (14.5)
Occupational status:	
Active	108 (32)
inactive	230 (68)
Medical coverage:	
None	66 (19.6)
RAMED/AMO ‘Solidarity’	130 (38.6)
AMO	141 (41.8)
Poverty status:	
Yes	126 (37.3)
No	212 (62.7)
Educational level:	
Unschooled	164 (48.5)
Primary school	65 (19.2)
Secondary school	80 (23.7)
University	29 (8.6)

IQR: interquartile range, RAMED: the medical assistance plan, AMO: obligatory health insurance, N: number, and %: percentage.

**Table 2:** Clinical and therapeutic characteristics.

Variables	Participants n = 338 (N%)
Duration of diabetes:	
<=10	175 (51.8)
>10	163 (48.2)
HbAc1 level (%)	
<7 optimal	51 (15.1)
7–9 suboptimal	238 (70.4)
>9 higher risk	49 (14.5)
Chronic complications:	
Yes	173 (51.2)
No	165 (48.8)
Comorbidity:	
Yes	137 (40.5)
No	201 (59.5)
Hypertension comorbidity:	
Yes	108 (32)
No	230 (68)
Obesity status:	
Yes	22 (6.5)
No	316 (93.5)
Smoking status:	
Yes	26 (7.7)
No	312 (92.3)
Adherence to treatment:	
Low	18 (5.3)
Moderate	95 (28.1)
High	225 (66.6)
frequency of medical visits per year:	
>4 visits	6 (1.8)
4 visits (recommended)	165 (48.8)
<4 visits	167 (49.4)
Treatment type:	
Insulin alone	155 (45.9)
Oral antidiabetics	135 (39.9)
Insulin + Oral antidiabetics	48 (14.2)
Traditional treatment:	
Yes	59 (17.5)
No	279 (82.5)
Specialty of treating physician:	
Generalist	170 (50.3)
Specialist	128 (37.9)
Both	40 (11.8)

HbA1c: glycated hemoglobin, N: number, and %: percentage.

**Table 3:** Quality of life for the participants (n = 338).

Dimensions	Items	Min–Max Scores	All Participants	Poverty Status		*p*-Value	Cronbach’s α
				Yes	No		
Energy and mobility	15	3.33–100	48.3 [33.3–70]	46.7 [35.3–65]	50 [31.7–71.7]	0.207 *	0.92
Diabetes control	12	0–100	62.5 [50.5–75]	75 [62.5–81.3]	58 [47.4–66.7]	<0.001 *	0.86
Anxiety and worry	4	0–100	81.3 [56.3–93.8]	93.8 [81.3–93.8]	68.8 [43.8–87.5]	<0.001 *	0.83
Social limitations	5	0–100	25 [10–35]	25 [10–35]	30 [15–40]	0.097 *	0.70
Sexual functioning	3	0–100	25 [0–64]	25 [0.41–7]	41.7 [16.7–75]	<0.001 *	0.94
Overall perceived QoL **	1	0–100	50 [25–50]	25 [25–50]	50 [25–50]	0.011 *	--
Diabetes severity ***	1	0–100	75 [50–100]	75 [75–100]	75 [50–100]	0.776 *	---

QoL: quality of life, * Mann–Whitney *U* test, ** low score indicates poor QoL, *** a high score indicates disease severity.

**Table 4:** Univariate linear regression of predictors of quality of life in diabetic patients.

Variable	Univariate Linear Regression								
	Diabetes Control			Anxiety and Worry			Overall Perceived QoL		
	β	95% CI	*p*	β	95% CI	*p*	β	95% CI	*p*
Poverty status									
Yes–No	13.4	9.74, 17.01	**<0.001**	19.1	13.40, 24.80	**<0.001**	−6.45	−12.40, −0.45	**0.035**
Gender:									
Male–Female	−1.11	−5.01, 2.80	0.578	−8.18	−14.10, −2.22	**0.007**	−2.42	−8.43, 3.60	0.43
Age									
≥60 years–<60 years	2.07	−1.76, 5.89	0.288	1.21	−4.69, 7.10	0.687	−1.95	−7.84, 3.94	0.516
Living area:									
Urban–Rural	−3.35	−7.62, 0.93	**0.125**	−1.37	−7.97, 5.24	0.684	−0.44	−7.04, 6.17	0.897
Marital status:									
Single	Ref.	--	**--**	Ref.	---	--	Ref.	--	--
Married	−0.081	−6.29, 6.12	0.979	7.42	−2.14, 17.00	**0.128**	4.23	−5.39, 13.90	0.388
Divorced	9.844	0.47, 19.22	**0.04**	10.45	−3.98, 24.90	**0.155**	4.99	−9.55, 19.50	0.5
Widowed	1.625	−5.98, 9.23	0.674	15.21	3.51, 26.90	**0.011**	−0.44	−12.23, 11.40	0.942
Occupational status:									
inactive–active	6.62	2.61, 10.6	**0.001**	18.7	12.8, 24.7	**<0.001**	0.21	−6.05, 6.47	0.948
Medical coverage:									
None	Ref.	---	--	Ref.	----	---	Ref.	--	--
RAMED/AMO ‘Solidarity’	1.47	−3.52, 6.46	0.562	3.11	−4.51, 10.72	0.423	−3.80	−11.84, 4.24	0.335
AMO	−10.52	−15.44, −5.59	**<0.001**	−17.28	−24.79, −9.76	**<0.001**	5.39	−2.55, 13.33	**0.182**
Educational level:									
Unschooled	9.77	2.86, −16.7	**0.006**	36.3	26.30, 46.30	**<0.001**	−2.75	−13.60, 8.04	0.616
Primary school	7.49	−0.18, 15.20	**0.056**	33.3	22.20, 44.40	**<0.001**	−8.67	−20.60, 3.29	**0.155**
Secondary school	3.16	−4.28, 10.60	0.404	22.8	12.10, 33.60	**<0.001**	−2.02	−13.60, 9.60	0.733
University	Ref.	--	--	Ref.	--	--	Ref.	--	**--**
Duration of diabetes:									
>10 − <=10	−1.67	−5.47, 2.12	0.386	−3.52	−9.35, 2.31	**0.236**	−2.54	−8.38, 3.30	0.393
HBAc1 level (%)									
<7	Ref.	--	--	Ref.	--	--	Ref.	--	--
7–9	3.5	−1.87, 8.87	**0.201**	6.39	−1.81, 14.60	**0.126**	−1.02	−9.28, 7.25	0.809
>9	0.79	−6.17, 7.75	0.823	15.24	4.61, 25.90	**0.005**	−7.42	−18.14, 3.29	**0.174**
Chronic complications:									
Yes–No	9.07	5.41, 12.70	**<0.001**	12.7	6.99, 18.40	**<0.001**	−8.62	−14.40, −2.85	**0.004**
Comorbidity:									
Yes–No	3.86	0.20, 7.70	**0.049**	6.29	0.38, 12.20	**0.037**	1.05	−4.90, 7.00	0.728
Hypertension comorbidity:									
Yes–No	3.36	−0.69, 7.41	**0.104**	0.3	−5.96, 6.56	0.925	4.89	−1.35, 11.10	**0.124**
Obesity status:									
Yes–No	5.9	−1.76, 13.60	**0.131**	−4.93	−16.80, 6.90	0.413	−10.20	−22.00, 1.60	**0.09**
Smoking status:									
Yes–No	−2.76	−9.88, 4.35	0.445	−3.53	−14.5, 7.43	0.527	−10.40	−21.30, 0.48	**0.061**
Adherence to treatment:									
Low	−0.82	−9.34, 7.70	0.850	−1.19	−14.31, 11.90	0.858	−3.39	−16.50, 9.68	0.61
Moderate	−3.25	−1.01, 7.50	**0.135**	5.75	−0.80, 12.30	0.085	−7.61	−14.10, −1.09	**0.022**
High	Ref.	---	**--**	Ref.	---	**--**	Ref.	--	**--**
Frequency of medical visits:									
>4 visits	Ref.	--	**--**	Ref.	--	--	Ref.	--	**--**
4 visits (recommended)	−5.21	−9.00, −1.42	**0.007**	−5.51	−11.40, 0.36	**0.066**	5.92	0.06, 11.80	**0.048**
<4 visits	−5.13	−19.48, 9.22	0.482	1.53	−20.70, 23.76	0.892	15.99	−6.17, 38.20	0.157
Treatment type:									
Insulin alone	−2.72	−6.82, 1.38	**0.193**	−5.49	−11.80, 0.81	0.087	3.69	−2.62, 10.00	0.251
Oral antidiabetics	Ref.	--	**--**	Ref.	--	--	Ref.	--	--
Insulin + Oral antidiabetics	−2.09	−7.94, 3.76	0.483	−5.63	−14.60, 3.36	**0.219**	5.38	−3.63, 14.40	**0.241**
Traditional treatment:									
Yes–No	7.08	2.14, 12.00	**0.005**	3.16	−4.53, 10.80	0.419	1.24	−6.45, 8.94	0.751
Specialty of treating physician:									
Generalist	Ref.	--	--	Ref.	--	--	Ref.	--	---
Specialist	−6.58	−10.60, −2.55	**0.001**	−13.23	−19.40, −7.10	**<0.001**	3.75	−2.52, 10.00	**0.24**
Both	−2.61	−8.65, 3.33	0.397	−8.36	−17.60, 0.84	0.075	4.34	−5.09, 13.80	0.366

β value: standardized beta-coefficient, CI: confidence interval, *p*-value in bold: significant value with a standard significance level set at 0.25 and Ref.: reference level.

**Table 5:** Multivariate linear regression of predictors of quality of life in diabetic patients.

	Multivariate Linear Regression			
Models	Sociodemographic, Clinical, and Therapeutic Predictors	β	95% CI	*p*-Value
Diabetes control	Poverty status	8.90	4.82, 12.97	<0.001
	Medical coverage (AMO)	−6.69	−11.92, −1.47	0.012
	Chronic complications	5.82	1.98, 9.67	0.003
	Obesity status	7.52	0.14, 14.90	0.046
Anxiety and worry	Poverty status	13.95	8.12, 19.78	<0.001
	Occupational status (inactive)	12.34	5.07, 19.60	<0.001
	Educational level			
	Unschooled	20.82	9.45, 32.18	<0.001
	Primary school	21.48	10.21, 32.76	<0.001
	Secondary school	19.21	8.77, 20.65	<0.001
	HbA1c (%) (>9)	9.49	0.25, 18.74	0.044
	Chronic complications	6.17	0.58, 11.75	0.031
	Duration of diabetes (>10 ans)	−6.72	−13.55, −1.89	0.010
Overall perceived QoL	Chronic complications	−7.50	−13.78, −1.22	0.019

β value: standardized beta-coefficient, CI: confidence interval.

## Data Availability

The data utilized and/or examined in this study are available from the corresponding author upon reasonable request.

## Abbreviations

QoL	Quality of Life
HbA1c	Glycosylated Hemoglobin
HRQoL	Health-Related Quality of Life
AMO	Obligatory Health Insurance
RAMED	Medical Assistance Plan
